# CRISPR mediated PRRS resistant pigs: biological success, welfare implications, and ethical regulatory challenges for sustainable swine production

**DOI:** 10.1186/s40813-026-00518-0

**Published:** 2026-06-12

**Authors:** Shahrukh Khan, Zeenat Korai, Shakal Khan Korai, Shengnan Li, Liting Yang, Safeer Ahmed, Seyidov Mirvasif, Anvarjon Kholmatov, Abdimumin Amirov, Shodi Xolov, Mohd Asif Shah, Xiaoshan Wang

**Affiliations:** 1 College of Animal Science and Technology, Yangzhou University, Yangzhou, 225009 P.R. China; 2 Jiangsu Yancheng Wetland Rare Birds National Nature Reserve, Yancheng, 320900 P.R. China; 3 Department of Veterinary Medicine, Faculty of Natural Sciences and Agriculture, Nakhchivan State University, Nakhchivan, AZ7012 Azerbaijan; 4 Laboratory of Biotechnology, Scientific-Research Institute of Livestock and Poultry, Institut Str., Kibray District, Tashkent Region, 111212 Uzbekistan; 5 Department of General Zootechnics and Veterinary Medicine, Tashkent State Agrarian University, 2A Universitet Str., Kibray District, Tashkent Region, 100700 Uzbekistan; 6 Kardan University, Parwane Du, Kabul, 1001 Afghanistan

**Keywords:** Porcine reproductive and respiratory syndrome, CRISPR-Cas9, CD163, Disease resistance, Pig welfare, Pig health management, Regulations

## Abstract

Porcine reproductive and respiratory syndrome (PRRS) remains one of the most significant health and welfare challenges in global pig production. The disease is associated with substantial economic losses, impaired herd health, increased antimicrobial use, and ongoing animal welfare concerns. Despite widespread implementation of vaccination, biosecurity, and herd stabilization strategies, PRRS continues to persist endemically in many production systems, highlighting the need for additional disease-control approaches. Recent advances in genome editing have enabled the development of host-directed resistance strategies in pigs. In particular, targeted editing of the scavenger receptor CD163, a key host factor required for PRRS virus entry into macrophages, using CRISPR–Cas9 has demonstrated resistance to several PRRSV strains in experimental models. Deletion of the SRCR5 domain of CD163 prevents viral infection while largely preserving normal receptor function, representing a more targeted approach compared with complete gene knockout. Current evidence suggests resistance to both major PRRSV genotypes, although most data derive from controlled experimental studies rather than commercial field conditions. Important considerations remain regarding long-term health and welfare outcomes, immune competence under production environments, ethical considerations surrounding germline genome editing, and differences in regulatory frameworks that may influence adoption. From a pig health management perspective, genome-edited PRRS resistance should be viewed as a complementary strategy rather than a replacement for established control measures such as vaccination, biosecurity, and herd health management. Careful evaluation, veterinary oversight, and coordinated regulatory guidance will be necessary to determine how this technology may be integrated responsibly into future PRRS control programs.

## Background

Pig production is a critical component of global food security, supplying affordable, nutrient-dense animal protein and supporting livelihoods across diverse production systems. Pork remains one of the most consumed meats worldwide, and agricultural outlooks continue to project sustained demand for pig meat, particularly in low- and middle-income regions where dietary patterns are shifting and urbanization is expanding [1, 2]. These tendencies provide an insight into the necessity of productive and sustainable swine operations that can address increased animal welfare, environmental stewardship, and responsible use of antimicrobials [3, 4]. Therefore, it is necessary to control disease in pigs not only to make a profit but also to ensure animal welfare, antimicrobial stewardship, and food-chain stability [5].

**Fig. 1:**
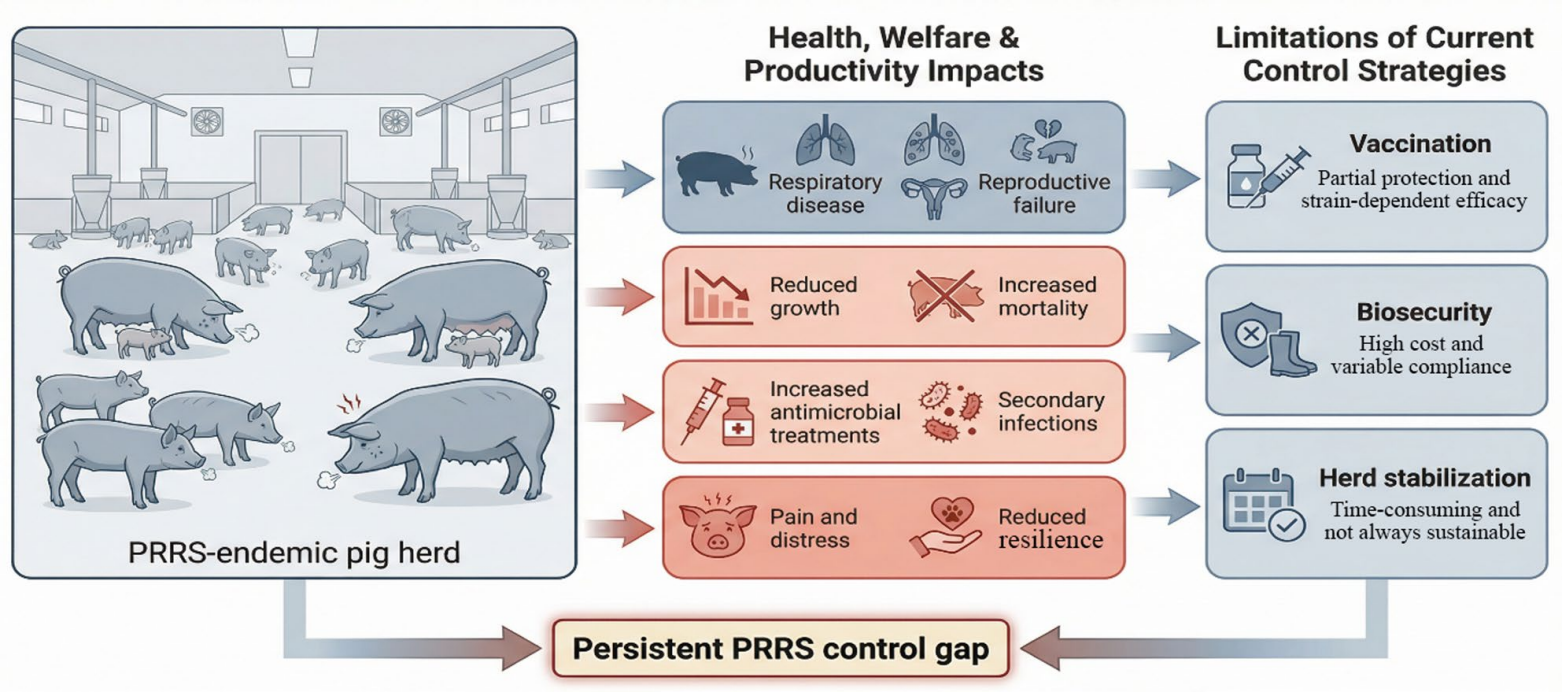
Conceptual summary of the impacts of porcine reproductive and respiratory syndrome (PRRS) on herd health, productivity, antimicrobial use, and animal welfare, and the limitations of current control strategies that contribute to a persistent control gap in endemic pig production systems

Among infectious diseases affecting pigs, PRRS remains one of the most significant and persistent challenges to herd health worldwide. PRRS affects pigs across production stages, typically presenting as reproductive failure in breeding herds and respiratory disease in nursery and grow-finish pigs [6]. At the herd level, outbreaks increase piglet mortality, reduce growth rate, decrease feed efficiency and delay market time. These issues cause financial losses and overworking of farmers and vets to recur [7, 8]. Due to the frequent endemics and inability to eliminate PRRS within production networks, the disease is considered a priority issue on regional and farm types [9] (Fig. 1).

Porcine reproductive and respiratory syndrome virus (PRRSV) biology contributes directly to these challenges. The virus primarily targets macrophages and modulates innate and adaptive immune responses, which can impair viral clearance and contribute to prolonged infection dynamics in affected populations [10, 11]. This causes the pigs to become susceptible to bacterial and comorbid respiratory diseases increasing the complexity of clinical manifestations and response to common therapeutic interventions [12]. As a result, outbreaks tend to stimulate the increase of antimicrobial use, particularly in nursery and grow-finish populations where co-infections frequently occur [13, 14]. Though there are many factors that determine the use of antibiotics, the major role of stewardship is controlling PRRS. This reduces immunosuppression and respiratory disease and thereby lessens and less severe antimicrobial therapy [15, 16]. In addition to viral determinants, host immune activation can also influence susceptibility to PRRSV infection. Activation of macrophages by bacterial components such as lipopolysaccharide (LPS), or by co-infections with other respiratory pathogens, can modulate the expression of receptors involved in PRRSV entry, including CD163 [17–19]. This interaction between inflammatory stimulation and receptor expression may partly explain the variability in disease severity observed among herds exposed to different pathogen pressures and environmental stressors. In commercial pig production systems where polymicrobial respiratory disease complexes are common, such host-pathogen interactions may significantly influence PRRSV transmission dynamics and clinical outcomes [13, 14, 20].

From an animal welfare perspective, PRRS is commonly associated with fever, lethargy, respiratory distress, reduced vitality, and elevated morbidity and mortality especially in young pigs and in herds experiencing severe clinical expression [21, 22]. The indirect impact is also on welfare, the more handling, repetitive treatment, segregation, and supportive care, the more stress increases. Welfare burdens are increased by secondary infections and retarded growth [23]. In the modern swine systems, the evaluation is not only based on the productivity but also on welfare and broader sustainability objectives [3, 24, 25].

Despite sustained control efforts, PRRS management remains imperfect. Vaccination particularly with modified live virus (MLV) vaccines remains a cornerstone of PRRS control, yet field protection is often variable and incomplete due to extensive viral genetic diversity and rapid PRRSV evolution [26]. Research indicates that cross strain protection is not always effective and varies in relation to the lineage of the virus, history of herd immunity and farm conditions [26]. The recombination between lineages such as its involvement with strains of MLV has only grown, complicating outbreak investigations and control strategies [26, 27].

Biosecurity and herd management are indispensable components of PRRS control, but they are operationally demanding. PRRSV can be introduced through multiple pathways (e.g., animal movements, contaminated fomites, personnel and transport networks, and potentially aerosol spread), and risk profiles vary substantially across regions and production system structures [28]. Nevertheless, extensive studies have demonstrated that science based biosecurity programs can reduce PRRS in breeding herds which are clearly beneficial but necessitate continuous investment and adherence [28, 29]. The virus can still reoccur or persist even with good systems, which is why the management of PRRS is a permanent affair, not a one-time solution [30].

Taken together, PRRS’s persistent burden and the limitations of existing tools highlight the need for novel, complementary disease control strategies that strengthen rather than replace current PRRS management programs (Fig. 1). Strategies to reduce pig vulnerability, host-based strategies, would enhance herd stability, alleviate welfare issues and support antimicrobial stewardship [10]. Genome editing and, in particular, CRISPR-based intervention targeting host factors that play a role in PRRS resistance can be effective in enhancing pig disease resistance [31, 32]. Host-directed resistance target host-virus interactions that are essential for viral entry or replication. This would reduce dependence on the strain-stressed vaccines in a heterogeneous viral environment [33]. However, from a pig health management perspective, the practical value of CRISPR based PRRS resistance will depend not only on biological effectiveness under controlled challenge conditions, but also on long-term health and welfare outcomes under commercial conditions, the integrity of surveillance and biosecurity programs after adoption, and regulatory feasibility across regions that influence implementation, trade, and public acceptance [34, 35].

## Genome editing as an emerging tool in pig health management

### Overview of CRISPR-Cas genome editing in pigs

Genome editing refers to a group of technologies that allow precise modification of an animal’s DNA with high accuracy and precision. CRISPR-Cas systems have become the most popular of these since they are relatively straightforward, efficient, and flexible in comparison to previous genome editing systems (e.g. zinc-finger nucleases or TALENs) [32, 36]. CRISPR-Cas genome editing has been used to edit genes linked to disease susceptibility, production traits, and biomedical interest in pigs and this has shown that it is technically feasible in a variety of applications [35, 37].

Considering the pig health management, one of the main characteristics of CRISPR based editing is that it is able to insert specific changes without the need to include foreign genetic material. Numerous disease resistance technologies are based on slight deletions or alterations in endogenous genes, and the resulting animals have genetic alterations that could be indistinguishable to the variants found in nature at the DNA sequence level [38]. This is what makes genome editing different than previous transgenic methods and has led to an increase in the excitement over its potential to be used in the livestock health and welfare enhancement.

Notably, the CRISPR-mediated edits could be structured in such a way that they do not interfere with normal physiological activity but instead disrupt pathways that are necessary to get infected by a pathogen. This precision is particularly important in veterinary applications, where unintended effects on growth, reproduction, or immune function could compromise practical utility [39, 40]. Consequently, genome editing is no longer being debated as an alternative to traditional disease-controlling strategies, but rather as a biological complement that may be used to enhance the resilience of the herd level when part of a broader health management strategy.

### Why host-directed disease resistance is attractive for PRRS control

The existing PRRS control measures are mainly focused on the virus with the help of vaccination, biosecurity and management measures aimed at minimizing the exposure and spread. The methods are still important parts of PRRS control, but their efficacy is limited by the significant genetic diversity and rapid evolution of PRRS virus (PRRSV), which may lead to inconsistent vaccine efficacy and persistent endemic circulation [10, 41]. PRRS control has therefore largely focused on mitigation and stabilization and not elimination especially in areas where pigs are very numerous and the introduction of the virus through them is frequent.

Host-directed disease resistance is a complementary approach where the emphasis is laid on the pig as opposed to the pathogen. Instead of trying to balance control interventions to the changing virus, host-directed strategies seek to alter host variables that are fundamental to viral entry or viral replication. Interrupting these host-virus interactions can inhibit infection despite the variation in the strain of the virus [42]. The idea holds a special appeal to PRRS where elimination is hard and long term management of disease is the main goal in most of the production systems.

Host-directed resistance has a number of possible benefits to veterinarians and producers. Ensuring pig resistance to PRRSV may enhance the stability of herd, reduce the clinical manifestation of disease, and decrease the incidence of secondary infections which contribute to the emergence of antimicrobial use [43]. In comparison to pathogen targeted tools, host-directed strategies are less prone to being compromised by viral mutation alone, which is likely to provide a longer-lasting protection against time [44]. Notably, these strategies are not expected to substitute vaccination, biosecurity, or herd management, but should be used in combination with them as an integrated health strategy.

In the context of a pig health management strategy, host-directed genome editing should not be judged solely by its capability to prevent infection, but also by its potential effects on animal welfare, immune competence and performance in the long term in a commercial setting. The responsible integration would need the ongoing veterinary monitoring, monitoring and compliance to the accepted biosecurity and management practices. When these conditions are fulfilled, host-directed genome editing may be a new weapon in the improvement of resilience to PRRS in a disease where the disease is endemic and hard to manage by alone applying existing measures [34].

## Molecular basis of PRRS resistance via CD163 editing

### Role of CD163 in PRRSV infection

**Fig. 2:**
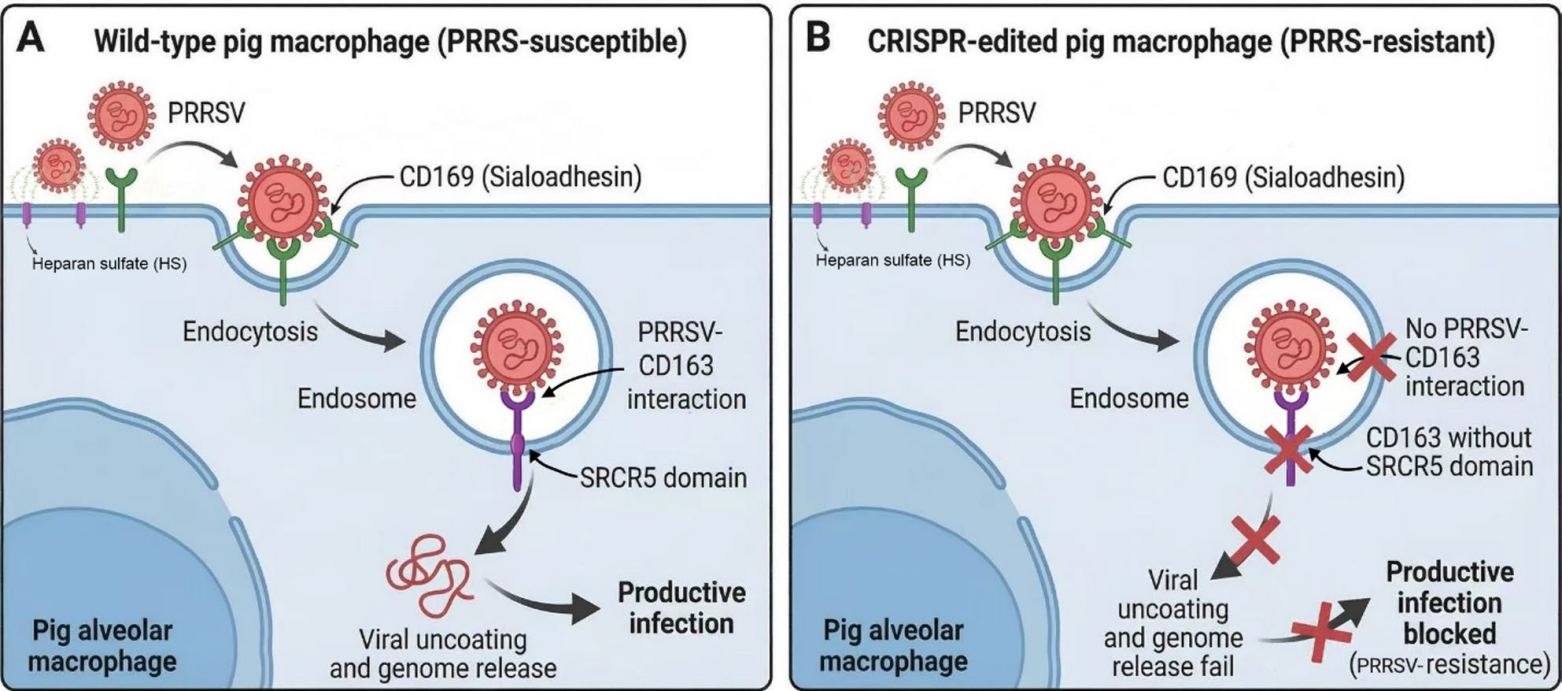
Mechanism of PRRSV entry and CRISPR-mediated resistance in pig macrophages. (**A**) In wild-type pig alveolar macrophages, PRRSV attaches to cell surface receptors such as heparan sulfate and CD169 (sialoadhesin), followed by receptor-mediated endocytosis. Within endosomes, interaction between PRRSV and the SRCR5 domain of the CD163 receptor enables viral uncoating and release of the viral genome into the cytoplasm, resulting in productive infection [52, 55–57]. (**B**) In CRISPR-edited pigs lacking the CD163 SRCR5 domain, PRRSV attachment and endocytosis may still occur; however, the absence of SRCR5 prevents interaction between PRRSV glycoproteins and CD163, blocking viral uncoating and genome release. Consequently, productive infection is prevented, conferring resistance to PRRSV [46, 58, 59]

PRRSV specifically infects porcine macrophages, especially those alveolar and other tissue-resident macrophage populations that play a critical role in innate immunity in the respiratory tract [45, 46]. One of the host factors that enable the PRRSV to replicate is the scavenger receptor CD163. This protein is located on the surface of these macrophages and is responsible for removing the hemoglobin-haptoglobin complexes and regulating immune responses [47, 48]. Various research studies indicate that CD163 is necessary to have a successful PRRSV infection. It has been shown through experiment that CD163 participates in key events of viral entry and uncoating in macrophages; inhibition of CD163 activity prevents productive infection [49, 50]. Experiments involving mapping identify a single location of the scavenger receptor cysteine rich domain 5 (SRCR5) as an important determinant of PRRSV permissiveness (Fig. 2). PRRSV attaches itself to SRCR5 in early infection and facilitates viral internalization, uncoating and replication [51, 52]. Infection does not necessarily require other CD163 domains and so SRCR5 is a good target to interventions that prevent viral entry without disrupting the overall roles of the CD163 in macrophages [53]. The expression of CD163 on macrophages is not static and can be influenced by inflammatory stimuli. Activation of macrophages by bacterial endotoxins such as lipopolysaccharide (LPS), or by co-infections with other respiratory pathogens, can modulate the expression of macrophage surface receptors including CD163 [17–19]. Increased receptor availability may enhance PRRSV entry and replication in macrophages, thereby facilitating infection [18, 54]. This mechanism may contribute to differences in disease progression observed under field conditions, where pigs are frequently exposed to multiple pathogens within the porcine respiratory disease complex (PRDC) [13]. Understanding these host–environment interactions is important when interpreting experimental infection studies and evaluating genome-editing strategies targeting CD163.

### CRISPR mediated CD163 editing strategies

Genome editing approaches that target CD163 have aimed at disrupting the virus-host interaction as opposed to the macrophage biology in general. Two major approaches have been followed: (i) total knockout of CD163, and (ii) domain specific editing where the SRCR5 region is deleted or edited but the rest of the receptor has been spared [50, 51].

PRRSV resistance can be completed with CD163 knockout having a strong resistance, which gives clear proof-of-concept evidence in host-directed disease control [60]. However, CD163 is implicated in the physiology and immunoregulation of macrophages, so, the deletion of the whole gene puts legitimate doubts on the immune competence, resistance, and lifespan health in the business setting [46, 61]. This is the reason why domain-specific editing is most desired now. The concept behind deleting SRCR5 is to maintain the essential activities of CD163 and delete the domain required to enter PRRSV so as to offer a solution that can satisfy both the animal welfare and production needs (Table 1) [46].

**Table 1:** Summary of CRISPR-edited PRRS pig studies targeting CD163

Study (Year)	CD163 editing strategy	Editing approach	Experimental model	PRRSV genotype tested	Main health outcome	Key implications for pig health management
United States (2016) [58]	Full CD163 knockout	CRISPR-Cas9 editing of embryos	In vivo pig challenge	PRRSV-2 (North American genotype)	No detectable infection under experimental PRRSV-2 challenge	Proof-of-concept that CD163 knockout can prevent PRRSV infection under experimental challenge; highlighted potential concerns regarding long-term immune function.
United Kingdom (2017) [46]	SRCR5 domain deletion (domain-specific)	Genome editing; macrophage assays	In vitro (porcine macrophages)	PRRSV-1 and PRRSV-2	Edited macrophages resistant to PRRSV-1 and PRRSV-2 infection with preserved biological function	Identified SRCR5 as the key domain required for PRRSV entry, supporting targeted CD163 editing strategies that preserve macrophage function.
United States (2017) [52]	SRCR5 replacement with human CD163-like domain	Genetic modification	In vivo and ex vivo	PRRSV-1 and PRRSV-2	Resistance to PRRSV-1 infection but susceptibility to PRRSV-2	Demonstrated genotype-specific differences in PRRSV susceptibility, highlighting the need to evaluate multiple viral strains when developing CD163-based resistance strategies
China (2019) [62]	SRCR5 deletion (exon 7 removal)	CRISPR-Cas9 embryo editing	In vivo pig challenge	PRRSV-1	No detectable infection under highly pathogenic PRRSV challenge	Confirmed the protective effect of CD163 exon 7 deletion in vivo using edited pigs and sibling controls, supporting translational potential of targeted CD163 editing
United States (2017) [63]	Maternal CD163 knockout	CRISPR-Cas9	In vivo reproductive model	PRRSV-2	No detectable fetal infection following PRRSV-2 challenge of CD163-knockout sows	Demonstrated that maternal CD163 knockout can prevent transplacental PRRSV transmission, highlighting potential benefits for reproductive herd health
China (2020) [64]	CD163 + pAPN double knockout	CRISPR-Cas9 + SCNT	In vivo pig challenge	PRRSV-2	Resistance to PRRSV and TGEV with reduced susceptibility to PDCoV	Demonstrated feasibility of engineering resistance to multiple viral diseases, highlighting potential for multi-disease genetic strategies and the need to evaluate broader immune consequences
United States (2024) [65]	Heritable SRCR5 deletion	CRISPR-Cas9	In vivo performance monitoring	Not always challenged	Heritable PRRSV resistance with normal growth and production performance reported	Addressed key pig health management concern by demonstrating heritable resistance without detectable impacts on growth or production performance

SRCR5 editing by CRISPR-Cas9 has been reported via the use of embryo based editing techniques (e.g. zygote microinjection or similar techniques) producing pigs expressing CD163 without SRCR5. These pigs develop freely in laboratory procedures, and show no sign of any apparent deficiency in baseline performance or general health and it can be concluded that SRCR5 targeting would confer protection with minimum side effects [65, 66]. However, there are PHM pertinent questions on to do with moderate immunological trade-offs, reactions to various co-infections, and lifelong performance in farm settings [30].

### Evidence for resistance across PRRSV genotypes

The main question of PRRS control is whether the resistance of CD163 can be useful within the genetic diversity of PRRSV strains that exist in commercial systems. It has long been demonstrated by experimental work that viral uncoating and replication in porcine macrophages is inhibited by editing of the SRCR5 domain of CD163 (invariably by deletion of exon 7), and can be effective in protective editing of PRRSV-1 and PRRSV-2 in controlled challenge models [46, 52, 58]. These papers presented that pigs that are devoid of SRCR5 are physiologically normal and fully resistant to infection in both in vitro macrophage cultures and in vivo challenge models.

Significantly, the evidence base was enhanced by the fact that the U.S. Food and Drug Administration (FDA) approved a deletion of exon 7 of the CD163 gene (CD163ΔE7) on April 29, 2025, to produce a deletion in the exon of a gene that causes pigs to be resistant to PRRSV (homoszygous animals). The FDA in its Freedom of Information Summary stated that the data provided justified molecular integrity of the edit, elimination of known and unwanted alterations by breed selection, animal safety, food safety and phenotypic and genotypic stability over generations. Viral challenge trials provided under the FDA package of effectiveness involved the use of a number of PRRSV-1 and PRRSV-2 isolates that represented the major lineages found in the American commercial population of pigs.

Majority of isolates that were used in these regulatory studies showed full resistance in homozygous edited pigs. Nevertheless, a single isolate of PRRSV-2, KS06-72109, exhibited partial protection, and some challenge experiments revealed that edited pigs were susceptible. The FDA evaluation showed that pigs that were carriers of CD163ΔE7 edit were resistant to all the tried strains but this isolate. The agency however determined that the practical risk of KS06-72109 was also low as the strain was not being circulated in the U.S. commercial swine population at levels that were appreciable when the strain was being approved.

The fact that this exception points to CD163-based editing is not yet a universally effective approach against all strains of PRRSV is worth mentioning. The available information, in its turn, suggests the possibility of the wider yet not entirely passive resistance that would imply the need to maintain a vigil on the viral diversity, engage in further studies of the challenge, and long-term monitoring of the potential viral adaptation or escape phenotypes as the technology has just entered the realm of the large-scale commercial exploitation.

In line with this interpretation, in vitro infection studies in macrophages and in vitro control experiments have shown that pigs edited at SRCR5 are resistant to infection by both PRRSV-1 and PRRSV-2 isolates [51, 66], but that regulatory challenge experiments in some individual isolates of PRRSV have shown resistance not be absolute (Table 1).

Across studies, SRCR5 edited pigs and their macrophages have shown key indicators of protection, including reduced or absent viremia, lack of characteristic clinical signs, and reduced transmission risk under experimental conditions [46, 65]. These results are firm proofs of the notion that host-directed gene editing can provide actual, biologically meaningful resistance. However, the majority of challenge experiments have been restricted to controlled experiments with limited number of virus strains and limited time. The largest unknowns in pig health management are: does the protection persist following repeated exposures, how these pigs perform and behave in the normal commercial environments and whether the virus evolves or does the ecological dynamics of the virus evolve [67, 68].

## Implications for pig health beyond PRRS resistance

Although the creation of PRRSV resistance is the main goal of CD163 genome editing, the applied importance of this practice to pig health control requires results that are not limited to the development of resistance to the virus. One of the main issues relevant to veterinarians and producers is whether genome-edited pigs can stay healthy, productive, and robust in the challenging environment of commercial farms, including co-infections, environmental stress, and divergent practices of their management of the latter [69, 70]. This part is hence dedicated to performance, reproduction, immune competence, and possible undesirable consequences which are the core of adoption in real world herd health programs.

### Growth performance and reproductive health

Available studies of CD163-edited pigs particularly those involving SRCR5 domain deletion generally report normal growth and baseline health under controlled conditions, with no obvious deficits in development or gross productivity traits compared with non-edited controls [65]. In the case of the reproductive and production outcomes, the results did not indicate any consistent negative impacts on the fertility or viability. This confirms the notion that domain-specific editing is able to preserve normal physiological functionality and inhibit the entry of PRRSV [69]. Medically speaking, this is an encouraging result: it indicates that PRRS resistance is possible without a sacrifice in core production characteristics, at least within the limits of controlled trials [33].

Most performance evaluations have been conducted in research environments with high biosecurity and limited stressor diversity, which may not reflect commercial conditions where thermal stress, dietary variation, social stress, and endemic co-infections are common [70]. These factors may have an insidious interaction with host genetics and are difficult to identify in small experimental samples. Moreover, there is a tendency towards the exposure of pigs to multi-pathogen respiratory disease complexes in farm systems. The interactions between PRRSV and bacterial pathogens determine the overall health outcome, which is dependent on the management practices [71]. Thus, the conclusions concerning growth and reproductive soundness cannot be discussed as final until they are supported by larger scale and longer period experiments under farm conditions [72].

### Immune competence and macrophage function

CD163 is a scavenger receptor expressed on macrophages that plays an important role in immune regulation and in the clearance of hemoglobin-haptoglobin complexes. Due to this, the CD163 modification casts doubts on the immune competence [61, 73, 74]. Recent research indicates that domain-specific editing is more appealing than complete gene knockout by showing that it is possible to inhibit PRRSV infection by targeting the SRCR5 domain alone without disrupting vital macrophage functions. Macrophages of SRCR5-edited pigs in laboratory experiments resist PRRSV without affecting normal macrophage biology. The studies with live animals challenge were also unable to identify an increased risk of common complications over the course of the observation [46, 66]. To address pig health issues, maintenance of effective innate immunity is required in the environment where respiratory pathogens are prevalent [75].

The immune competence depends on the recurrence of exposure to the pathogen, chronic stress, and nutrition or environmental constraints. There is limited evidence regarding long-term immune stability, balance of inflammation and response to co-infections in commercial environments [34]. The broader role of CD163 in immune regulation is also subject to small adjustments in the signaling, and it can only reveal itself when under sustained multi-pathogen pressure or during stressful phases such as late pregnancy and lactation in sows [76]. These uncertainties justify longitudinal immune monitoring and farm-based health surveillance in any translational pathway for PRRS-resistant pigs.

### Potential unintended biological effects

Off target editing remains a recognized concern in genome editing, although advances in guide design, nuclease engineering, and validation strategies have improved specificity and reduced risk [32, 77]. The majority of published CD163-editing literature contain targeted molecular validation of the desired edit and screen of significant unintended edits, however, whole-genome-wide evaluation is not consistently reported and reporting criteria vary by study and jurisdiction [31]. Genomic stability is relevant to PHM, as the undetected off-target events have the potential to affect health, welfare, or production outcomes, particularly across generations.

Even specific modifications can carry pleiotropic effects in case the desired genes are more generally biological. Although large adverse events have not been reliably documented in CD163-edited pigs, a negative result is not a positive result, especially when it comes to large sample size or a lengthy follow-up duration to detect an adverse event [78]. The possible system-level effects may be a change in the reaction to stressors, a variation in the predisposition to non-PRRS pathogens, or alterations in the inflammatory equilibrium, which is extremely important to veterinarians who deal with multifactorial herd health challenges [79, 80].

The gaps that have the most significant consequences to the overall health management of pigs are associated with the long-term and multi-generational outcomes in commercial settings. Information is still scanty regarding lifelong productivity, sow survival, piglet resilience, and herd level interactions in endemic disease settings [81]. To solve these gaps, standardized phenotyping, long-term welfare and health surveillance and farm-scale analyses will have to be incorporated in the current herd health programs and surveillance systems [82]. These steps are essential before genome-edited PRRS resistance can be responsibly considered for widespread adoption in commercial pig production.

## Animal welfare implications in commercial pig production

Animal welfare is inseparable from pig health management, and any novel disease-control intervention should be evaluated in terms of how it changes the lived experience of pigs in commercial systems. PRRS is associated with prolonged illness, respiratory compromise, increased mortality, and major disruptions to routine management, all of which carry direct and indirect welfare costs [7, 8]. Disease resistance therefore has the potential to meaningfully improve welfare, but the magnitude of benefit will depend on how genetic resistance is integrated into broader herd health strategies rather than treated as a standalone solution [83, 84]. From a PHM perspective, welfare evaluation must be linked to practical decision-making: whether the intervention reduces morbidity, improves robustness, and supports sustainable, welfare positive management.

### Potential welfare benefits of PRRS resistance

PRRS resistant pigs are expected to be protected from the clinical manifestations of PRRSV infection, including fever, lethargy, respiratory distress, impaired growth, and in breeding systems, reduced reproductive performance. Morbidity and mortality may be prevented at the biological level, which will reduce infection in young pigs, thus enhancing the welfare of herd and decreasing the number of clinical interventions that will have to be repeated [85, 86]. Controlled challenge studies have demonstrated that CD163-edited pigs are highly resistant and have lower clinical and pathological outcomes than controls, which confirms the potential of a significant welfare benefit in the presence of PRRS pressure [87].

PRRSV induced immunomodulation can predispose pigs to secondary infections and complex respiratory disease, often increasing treatment intensity in affected populations [88]. The decrease in PRRSV susceptibility would have an indirect benefit on reducing the use of antimicrobials by reducing the incidence and intensity of respiratory disease episodes which lead to therapeutic interventions. This is in line with the aims of antimicrobial stewardship and it could help to lessen welfare costs relating to repetitive handling, injections, and adverse treatment impacts [12, 89]. Although the use of antimicrobial is complex, it is generally viewed that PRRS control is an enabling factor in swine systems stewardship due to its potential to lower the underlying disease pressure that spurs the need of treatment [13].

Disease resistance may contribute to improved robustness by reducing physiological stress, inflammation, and the immune disruption associated with PRRSV infection. Pigs which can escape significant infectious issues might have better adaptation to the environmental and managerial stressors, which can enhance welfare during the production cycle [90]. However, robustness should be assessed holistically and not inferred solely from the absence of a single disease, especially in commercial settings where multiple pathogens and stressors interact [22].

### Risks of welfare trade-offs

A key welfare concern is that genetic resistance could be perceived as a substitute for good husbandry. Any benefits to the welfare would be diluted, or even lost, should PRRS resistance make farmers reduce housing quality, stocking density, ventilation or other health-supportive measures. This threat of technological replacement is significant since the disease resistance cannot be used as an excuse to implement practices that lower the level of comfort, behavioral expression, or quality of the environment [86]. Resistance in the context of pig health management can be regarded as a premium to high standards of care, and not an authorization to inferior levels of care.

PRRS resistance could also reduce perceived risk and contribute to complacency in biosecurity and disease surveillance. A decrease in vigilance can increase the risk of introducing or spreading other pathogens, which can negatively affect the welfare and health of herd [28]. Also, PRRSV control is not always a stand-alone respiratory disease approach; when attention is no longer given to integrated prevention, the overall welfare of the herd could decline despite PRRS control [22, 68]. Thus, the veterinarians have a significant role in ensuring that resistance is incorporated into coordinated programs of herd health and that surveillance and biosecurity is robust [91].

### Welfare assessment under farm conditions

Most welfare relevant observations for CD163-edited pigs have been generated in research settings, and data from commercial environments are limited. An effective welfare test must then utilize animal-based validated measures of welfare in farm conditions: respiratory effort, coughing, body condition, lameness scoring, injury prevalence, behavioral time budgets, and mortality or removals. These are to be accompanied by production measures, which indicate chronic health issues, including growth variability and days to market [92, 93]. With these indicators in normal herd surveillance, the veterinarians are able to tell whether genetic resistance is indeed a good thing in actual practice.

The response of welfare to change is time dependent and may be affected by cumulative stressors, seasonal variation and the exposure to multi pathogens. Genome editing, particularly delayed or context-specific, which can be difficult to detect in short-term studies, must be monitored over the long-term, e.g., minor immune dysfunction, disease susceptibility, or stress-sensitivity [94]. Continuous welfare tests help producers and veterinarians to discover the unforeseen results, early enough and in a manner that the introduction of genetic resistance is performed in a responsible, welfare-enhancing management system.

## Ethical considerations relevant to pig health practice

Ethical considerations are increasingly recognized as an integral component of pig health management, particularly when new technologies have long-term and heritable effects. For veterinarians and producers, ethical evaluation is not an abstract exercise: it shapes responsible use, influences public trust and market access, and can determine whether a technology gains or losses “social license” in commercial pig production [68, 95]. Because PRRS-resistant pigs created through genome editing are intended for deployment within food systems, ethical analysis must explicitly connect to pig health outcomes, welfare safeguards, and governance practices that are transparent to stakeholders [24, 96].

### Germline genome editing and responsibility in livestock

The mechanisms which mediate PRRS resistance by CRISPR are based on germline genetic editing, i.e. genetic changes are passed to the next generations. This is in contrast to the traditional veterinary treatment, which involves vaccines, antibiotic and biosecurity because it permanently alters the genes of the breeding herd and could spread widely through the genetic pathways [31]. The germline editing puts a higher demand on the quality of data and monitoring the long-term and accountability to pig health professionals. Early-life development and adoption choices may have long-term biological impacts on pigs and their health [97, 98].

Ethical acceptability in livestock production is often framed around proportionality and purpose: interventions are easier to justify when they clearly reduce disease burden and animal suffering and when potential harms are actively managed [99]. The expense of ill health can be excessive in the instance of PRRS respiratory distress, fever, death, secondary infections. The possible benefit of a resistance plan is welfare so long as it suffers a substantial decrease in clinical effects devoid of introducing new health risks [100]. However, proportionality however requires a sound argument that the editing will not have any lasting adverse effects on the immune competence, durability, or well-being particularly in commercial farms where animals are exposed to a variety of pathogens and other forms of stresses [101]. This renders it a plausible moral duty of veterinarians: in advising the producers they need to weigh the anticipated advantages against the unpredictables and take decision-making options which depend on the health consequences of animals as opposed to the existing economic pointers [97, 98].

### Instrumentalization versus welfare driven innovation

A recurring ethical concern in livestock biotechnology is instrumentalization treating animals primarily as tools to optimize production efficiency rather than as sentient beings whose welfare must be protected. Genome editing in farmed animals: Ethical reviews emphasize that the benefits in animal welfare should be real and quantifiable, and that technology should be used to achieve larger system objectives that would encourage high welfare standards, as opposed to intensification with no protection [102–104]. This matter gains particular significance in the PRRS setting when genetic resistance is offered as a solution to the systemic problems (including high disease pressure associated with the animal movement networks or environmental inefficiency), but not as a component of the comprehensive health management.

A welfare driven approach requires that genome editing be used to reduce suffering and improve health while maintaining good husbandry, biosecurity, and welfare supporting environments. The approach to ethical boundaries is when disease resistance is applied to reduce investment in ventilation, space, enrichment or staffing or used in a manner that undermines the motivation to continue with a robust preventive medicine program [68]. The PHM view on the ethical standard is that whether pigs, which can live on worse conditions when a disease is absent, would live on order to be better than the situation without the intervention is irrelevant; and if the intervention will increase the net welfare in the systems which also meet the right standards of welfare is what counts [101]. This puts the veterinarians at the center as a steward: they can contribute to the realization of genetic resistance being introduced as a complement, rather than a replacement of, biosecurity, welfare evaluation and herd health planning in general.

### Public acceptance and trust in pig health interventions

The key factor to include in the integration of genome edited pigs in the commercial production systems is public acceptance. The research has been uncovering continuously that the consumers are more favorable to the purpose and do-good gene-edited foods that they are confident of the governance, and that they are secured of the welfare and safety of the animal food regulation [105, 106]. During the study, the given applications are considered more positively when they are regarded to advance the well-being or prevent the disease than the ones that are perceived as motivated by the profit, and open communication can be a long route to make the application be accepted [107]. Nonetheless, the manipulation of animals over plants is more of a concern to the consumers, hence the importance of being in it and suitable assurances [108].

Trust is a direct influence of market access and long term viability of health innovations to producers and veterinarians. Lack of transparency or poor stakeholder engagement will ruin consent in the instance of scientifically confirmed safety and effectiveness [109, 110]. To have ethical governance therefore the following must be fulfilled (i) there must be a clear declaration of welfare goals, (ii) monitoring plans which can prove that there is a net benefit in welfare and (iii) communication to explain why editing was done and the way risks were managed [111]. Trust and adoption are also determined by real life regulatory milestones. This article that the regulators of the U.S. have awarded judgment upon pigs that contain the PRRS-resistant gene-editing has also provoked further concern with the responsibility and traceability requirements, along with good judgment usage [28, 112]. Veterinarians remain the key communicators in the evidence-to-practice translation process, through their responsibility to monitor and assure welfare as well as with excellent communication with the producers and the population.

## Regulatory and practical barriers to implementation

**Fig. 3:**
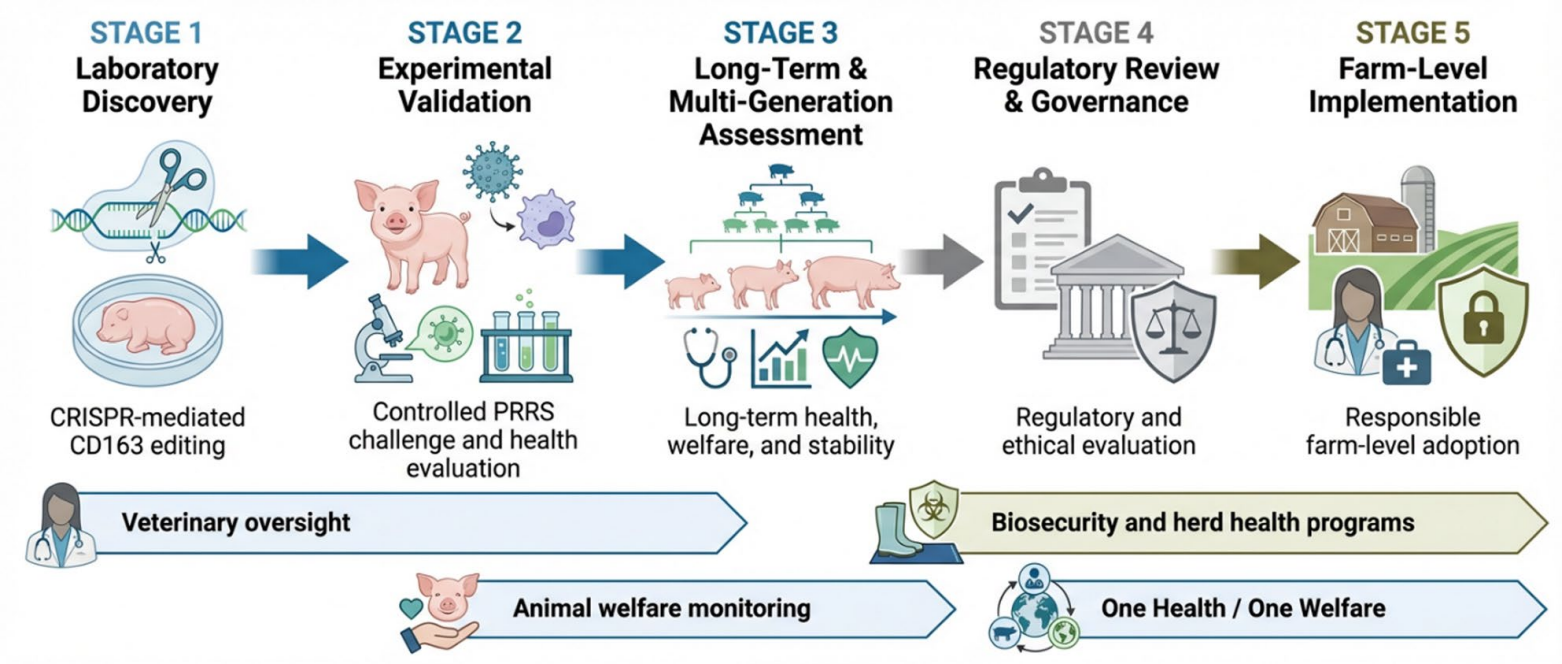
Roadmap from laboratory discovery to farm-level implementation of PRRS-resistant pigs

As much as CRISPR mediated PRRS resistance has shown high biological plausibility under controlled conditions, it is up to regulatory and governance structures to dictate eventual translation to commercial pig production. These frameworks determine to the veterinarians and producers whether genome edited pigs can be introduced to breeding or not, how they should be monitored, how the pork products are governed within the food chain, and whether international trade barriers can occur [113, 114]. The issue of regulatory divergence over scientific feasibility has become one of the leading limitations on the schedule of adopting PRRS resistant pigs [115] (Fig. 3).

### Regulatory status relevant to pig production

#### European Union (EU)

In the European Union, organisms that have been created using the specific mutagenesis technique, e.g. CRISPR-based genome editing, have traditionally been regulated under GMO legislation. This is after the Court of Justice of the EU (CJEU) in Case C-528/16 in 2018 perceived gene-edited organisms as GMOs under Directive 2001/18/EC with limited exemptions regarding older mutagenesis techniques. In the case of livestock, the GMO system will require a lot of evidence, lengthy approval processes, and increased political and market challenges. These restrictions may drastically restrict immediate-term business implementation, even in cases where edits are not injected with foreign DNA [116]. This poses a significant practical obstacle to the producers and veterinarians who wish to test farm-level integration in EU systems, in terms of pig health management. Without an effective and corresponding regulatory route, large-scale field implementation is challenging.

#### United States (USA)

In the United States, oversight of genome edited animals is centered on FDA regulation of Intentional Genomic Alterations (IGAs) in animals, with evaluation focused on animal health, food safety, and environmental considerations [117]. The agency has already provided finalized guidance on a risk-based approach and the process of approval of heritable IGAs [117, 118]. Although this route is more evident than the EU one, it is also resource intensive and needs a large evidence package and regulatory involvement. PRRS resistance has lately been made more tangible to the U.S. regulatory pathway. In April 2025, the FDA gave approval to a PRRS-resistant pig gene edit to be utilized in the U.S. food supply chain [119, 120]. In addition, the FDA approval dossier also has a valuable practical implication in the pig health management: the CD163ΔE7 edit was very resistant to the various PRRS strains, but not totally resistant to the PRRS-2 isolate of KS06-72109 in the provided effectiveness studies. The FDA found this acceptable because in part because the isolate was thought to be of low prevalence in the U.S. commercial population today but this finding highlighted that regulation approval does not remove the necessity of continuing post-deployment surveillance, strain follow-up, and veterinary control to identify possible variants of viral adaptation or escape. This regulatory breakthrough is a significant move towards making gene-editing technologies become useful to veterinarians and producers, but it is not the final part of the implementation process as it remains challenging to regulate the use of gene-editing technologies and find a balance between post-market monitoring and supply-chain management, and compatibility with non-U.S. export markets who might have a divergent regulatory framework on the use of gene-editing technologies [46, 58, 118, 121].

#### China

China is the world’s largest pork producer and has invested heavily in biotechnology to enhance food security and agricultural competitiveness. According to the recent policy indicators, there is an increased interest in the use of gene-editing technologies and breeding innovation, and it is reported that one of the priorities is to create high-performance animal and to increase domestic breeding capabilities of precision-breeding [122]. However, commercialization of gene-edited livestock is still very young and regulatory clarity of food animals might not be congruent with the better-established systems of those applied to crops [123]. This can have implications worldwide on pig-health wherein the innovation can initially be translated where and how PRRS-resistant pigs may manage to escape into commercial systems. This would create an asynchronous adoption of the leading pork-exporting and pork-importing regions [124].

### Food safety, traceability, and veterinary oversight

Food safety is a central requirement for adoption of genome edited pigs. Regulatory reviews usually determine the safety of meat of these animals to be consumed, and whether it is comparable to conventional products, and also determine any potential unintended impact that is plausible as relates to animal health or the composition of the food [125]. Traceability is even more complicated: the deletions in the SRCR5 gene like the deletion in the CD163 gene may not result in phenotypic markers, and the deletion can be detected based on the availability of genetic reference data and supply-chain controls. This makes the issue of transparent documentation, breeding dissemination governance, and where necessary traceability systems more significant to industry and regulators [126, 127].

Veterinarians are central to responsible implementation because they link innovation to real world herd health outcomes. Their roles include (i) evaluating and communicating evidence on health and welfare, (ii) ensuring that PRRS resistance is integrated with vaccination, biosecurity, surveillance, and herd stabilization plans, and (iii) supporting longitudinal monitoring for unexpected health outcomes or shifts in disease ecology. It is also essential that, in the post-deployment phase, genetic resistance does not unwittingly induce complacency in biosecurity and surveillance, which would pose an added threat of other pathogens and welfare damage, as a result of veterinary surveillance [68, 128].

### Why regulation is the main bottleneck for translation

From a pig health management perspective, the primary barrier to implementation of PRRS resistant pigs is increasingly regulatory divergence and uncertainty, rather than scientific feasibility alone. Although the biological nature of resistance has been confirmed in controlled studies, the lack of consistency in the regional approaches between process-based and product/risk-based approaches generates confusion among producers, veterinarians, and breeding companies with regard to investment decisions, field-trial design, and commercialization timelines [129, 130]. This ambiguity also makes international trade difficult: pork systems are internationally networked, and regulatory failure may result in market segmentation, labeling conflicts, or import bans, despite domestic approval being obtained [131].

Bridging the translation gap will require governance that is proportionate to risk, informed by evidence, and aligned with animal welfare objectives while maintaining transparency and public trust. In practice, this would mean that pre-marketing evidence packages would have clear requirements, that post-marketing surveillance must be conducted, that traceability is required where necessary, and that the incorporation of gene-edited pigs into current herd-health systems should be advised [113] (Fig. 3).

### Industry structure and dependence on breeding companies

The other pragmatic reason given as to why genome-edited PRRS-resistant pigs should be adopted is the possibility of concentration of innovation in the hands of a few large breeding companies that have the technological capability, intellectual property, and regulatory capabilities to create and commercialize genome-edited livestock [132–134]. The production systems of modern pig production have already been dominated by special breeding pyramids, where large numbers of multinational breeding organizations providing elite genetic lines to commercial producers [135, 136]. This framework could also be strengthened by the introduction of genome-editing technologies since the development, regulatory approval, and the dissemination of the edited animals would consume significant investments in biotechnology infrastructure, regulatory compliance, and extensive breeding projects [40]. Consequently, the availability of PRRS-resistant genetics could rely on collaboration with these genetics providers, which could also make large genetics providers more strategic to the swine industry.

This structural dependency evokes some considerations as far as pig health management is concerned. Regional regulatory systems, licensing arrangements, and supply-chain dynamics may lead to producers having unequal access to better genetics. Besides that, the localization of innovation among few organizations may affect how fast technological diffusion could be and the price of adoption by commercial farms [31, 83]. These issues will need to be tackled through open breeding dissemination policies, alignment of regulations and cooperation between breeding firms, veterinarians, farmers, and policy makers to enable the widespread and prudent application of the advantages of genome-edited disease resistance to a broad range of production systems.

## Future perspectives for pig health management

CRISPR-mediated resistance to PRRS will have potentials or not to enhance the pig health depending on its effectiveness in assessment, implementation, and regulation in relation to the already established herd health management systems. Scientific evidence of concept of CD163-based type of resistance is high, but the future of this field should not be limited to controlled challenge studies but should shift towards evidence application that can underpin day to day decision making by the veterinarians and the producers [137]. This would involve farm relevant validation, open surveillance and accountable governance models that uphold biosecurity, and welfare protection and trust in the society [81, 87].

### Research priorities

PHM relevant evidence must include comprehensive welfare assessment across the full production cycle, not only short term outcomes after PRRS challenge. The validated animal-based welfare measures, including respiratory effort scoring, injury prevalence, behavior time budgets, mortality/removal records, and recovery trajectories, should be applied in future research. These are to be accompanied by chronic stress and resilience measures under normal management conditions [23, 101]. Longitudinal welfare data is also critical to establish that early life losses of PRRS-related disease are converted into long-term welfare benefits via finishing and in the case of breeders, through several parities.

Because genome editing is heritable, multi-generation monitoring is critical for detecting delayed biological effects, confirming stability of PRRS resistance, and evaluating impacts on reproductive performance, robustness, and sow longevity outcomes that strongly influence herd level welfare and economics [43, 138]. Multi-generation testing is also used to determine whether the process of selection and propagation of edited lines has created unintentional trade-offs particularly when subjected to cumulative stresses commonly experienced in commercial production. The pragmatic solution is to incorporate multi-generation surveillance into the well-structured breeding programs based on standard phenotyping and regular veterinary checks [139].

Small experimental studies provide critical mechanistic insight but cannot fully represent the complexity of commercial pig production, including co-infections, seasonal stressors, variable nutrition, and management heterogeneity [140]. Large-scale trials in natural endemic settings are thus required to measure effects on clinical disease manifestation, productivity, antimicrobial consumption, and welfare consequences on a herd basis. These trials ought to have several production systems and geographic environments to enhance generalizability and permit effective estimation of actual effect sizes in the real world [141]. Especially, the farm-scale assessment is essential since the success of PRRS control is very systemic and heavily relies on the quality of surveillance and biosecurity practices implementation [142].

### Integration with existing PRRS control strategies

CRISPR mediated disease resistance must not be shown as a substitute to existing PRRS control strategy, but rather it should complement the known practice. It is necessary to vaccinate in order to reduce the disease severity, enhance the herd immunity, and pursue higher health goals. However, the effectiveness of vaccines cannot be always the same as PRRS is very genetically differentiated and can undergo evolution [143]. There is also a need of herd stabilization and biosecurity. The resistance of pigs to PRRS will not make the other pathogens disappear, besides the general respiratory illnesses and the augmentation of the welfare expenses can be raised through the oversights in the management [144].

For herd health programs, PRRS resistant pigs may help to enhance stability in the sense that the dynamics of clinical disease expression and transmission may be reduced; however, to continue to reap benefits in the long-term, high standards of management, monitoring, and surveillance should be maintained. Nevertheless, the long-term benefit consists in the maintenance of high management standards, being observed and monitored all the time. PRRS surveillance, outbreak investigation, and biosecurity audit, thus, should be instituted as an element of PHM implementation. This will prevent the effect of complacency wherein the perceived resistance will be utilized to the less prevention practice [145, 146]. The integration planning must clearly indicate how edited pigs can be implemented to fit the current control processes (e.g., gilt introduction, herd closure plans, vaccination programs, and diagnostic surveillance), to make sure that genetic resistance enhances instead of breaking the comprehensive health control [84].

### Role of veterinarians in responsible deployment

The key role in responsible deployment of genome edited pigs will be occupied by veterinarians since they have an interface between scientific innovation and farm practice. They must enlist the use of new evidence, coach producers on the benefits and the uncertainties, incorporate resistance in herd-health programs and monitor long-term health and welfare effects on a herd basis [147]. Veterinary supervision is also required to make genetic resistance under the administration of biosecurity and surveillance control measures to protect pigs against different pathogens and maintain the wellbeing of pigs is not undermined [148].

There are also regulatory trends that strengthen the veterinary role. The FDA guidance on heritable intentional genomic changes in animals issued finally points to a risk grounded model and protocol to navigate through gene altered animals, and the implication is on following anticipations and regulation that is necessary. With regulatory decisions being translated into actual world supply chains, veterinarians also can be viewed to play a central role in communicating to the producers, regulators and the population, and these factors are transparency and trust, which are essential influencing factors in adoption and market viability [67, 86]. Active participation of veterinarians can therefore help resolve the best practices which can then concentrate on the health, well-being and sustainability of animals such that should CRISPR mediated PRRS resistance be adopted, it can have net beneficial outcomes in commercial pig farming.

## Conclusions

CRISPR-mediated genome editing targeting the CD163 receptor represents a promising research approach for reducing the impact of PRRS in pig populations. Experimental studies indicate that targeted editing of the CD163 receptor, particularly within the SRCR5 domain, can confer resistance to several PRRSV strains under controlled experimental conditions. However, current evidence does not support assuming uniform resistance across all viral strains or production environments. From a pig health management perspective, genome-edited PRRS-resistant pigs should be considered a complementary tool rather than a replacement for established control strategies such as vaccination, biosecurity, surveillance, and herd stabilization. Effective disease management in commercial systems will continue to depend on integrated herd health programs that combine genetic, immunological, and management-based approaches.

The potential implementation of genome-edited PRRS-resistant pigs in commercial production will depend not only on demonstrated biological efficacy but also on continued evaluation of animal health, welfare, long-term performance, and regulatory acceptance. In addition, monitoring for potential viral adaptation and ensuring responsible breeding practices will be important considerations as the technology progresses. Overall, genome editing targeting PRRS susceptibility may provide an additional tool for improving pig health and reducing disease burden when applied cautiously within veterinary-led herd health programs. Continued collaboration among researchers, veterinarians, producers, breeding organizations, and regulators will be essential to ensure that developments in genome editing are evaluated carefully and integrated responsibly into modern swine production systems.

## Data Availability

No datasets were generated or analysed during the current study.
